# Smart Anomaly Detection and Prediction for Assembly Process Maintenance in Compliance with Industry 4.0

**DOI:** 10.3390/s21072376

**Published:** 2021-03-29

**Authors:** Pavol Tanuska, Lukas Spendla, Michal Kebisek, Rastislav Duris, Maximilian Stremy

**Affiliations:** Faculty of Materials Science and Technology in Trnava, Slovak University of Technology in Bratislava, 917 24 Trnava, Slovakia; lukas.spendla@stuba.sk (L.S.); michal.kebisek@stuba.sk (M.K.); rastislav.duris@stuba.sk (R.D.); maximilian.stremy@stuba.sk (M.S.)

**Keywords:** anomaly detection, industrial internet of things, industry 4.0, neural network, predictive maintenance, smart system, sound analysis

## Abstract

One of the big problems of today’s manufacturing companies is the risks of the assembly line unexpected cessation. Although planned and well-performed maintenance will significantly reduce many of these risks, there are still anomalies that cannot be resolved within standard maintenance approaches. In our paper, we aim to solve the problem of accidental carrier bearings damage on an assembly conveyor. Sometimes the bearing of one of the carrier wheels is seized, causing the conveyor, and of course the whole assembly process, to halt. Applying standard approaches in this case does not bring any visible improvement. Therefore, it is necessary to propose and implement a unique approach that incorporates Industrial Internet of Things (IIoT) devices, neural networks, and sound analysis, for the purpose of predicting anomalies. This proposal uses the mentioned approaches in such a way that the gradual integration eliminates the disadvantages of individual approaches while highlighting and preserving the benefits of our solution. As a result, we have created and deployed a smart system that is able to detect and predict arising anomalies and achieve significant reduction in unexpected production cessation.

## 1. Introduction

Dynamic developments in informatics and intelligent technologies have led to the fourth industrial revolution. The ideas of this concept have been promoted in industry, but the leading innovators include the automotive industry and companies that primarily exploit the potential of automation and robotics.

The basic components of the Industry 4.0 concept include, among others, Industrial Internet of Things (IIoT), wireless and intelligent sensors, big data, and artificial intelligence [1,2,3].

The development of advanced sensors allow the collection of huge amounts of data in real time, their processing and storing, followed by the analysis and discovering of new knowledge that can be used to predict the future behavior of industrial systems, including the identification and prediction of various anomalies.

This principle is also widely used in the process of production equipment maintenance and has led to the acceleration of a special concept called Maintenance 4.0 [4,5]. According to [6], it is important that the maintenance department has the tools and techniques at its disposal to ensure the ability to respond reliably to problems. The use of intelligent approaches such as IIoT, dig data, or artificial intelligence can effectively detect where faults occur. At present, it is no longer a problem to predict the behavior of individual systems in the production process. Therefore, it is not difficult to effectively plan preventive maintenance, thus minimizing arising failures.

One of the main tasks here is data which is actually information coming from the production process. Most of the equipment in current production lines are able to produce important information that can later be analyzed and the gained knowledge used more effectively. Using this knowledge, the standard preventive maintenance changes to predictive. This approach, often called Maintenance 4.0, can then more effectively address issues that arise, including those that are unknown in advance.

The basic elements of Maintenance 4.0 are defined in [7,8]. From our point of view, the area of predictive maintenance and trigger-based maintenance is particularly important. Predictive maintenance (PdM) is one of the key components of Maintenance 4.0. By its consistent use in manufacturing companies, it is possible to achieve a significant increase in the life of individual devices as well as a reduction in planned downtime, but especially unplanned ones [9,10].

According to [11], one of the key components of PdM is anomaly detection. Its principle is that it looks for anomalies in data or processes so that it can inform maintenance staff in advance of an emerging problem. The fact is that a large number of anomalies in the manufacturing process remains undetected and hidden from conventional detection techniques [12].

For this reason, it is not only possible to use standard tools and techniques, but it is necessary to focus on the development of unique approaches that can solve the problem [13,14,15]. It is always associated with a specific production process and specific problems that need to be solved in this way.

Our contribution is focused on solving issues that are associated with the production process in the automotive industry. Specifically, it is a conveyor used in the assembly process. The key issues that out paper addresses are:

- Assembly conveyor is a single point of failure that can cause assembly process cessation,
- Bearing failure can occur any time on the assembly line despite the standard preventive maintenance,
- Decrease the production downtime using the predictive maintenance approaches.

The assembly process is one of the most important processes in car production. Any problems that arise have a much greater impact on the flow of production than other processes. Any cessation of production, due to a problem in the assembly process, is quantified at such amounts of money that it does not leave any production manager calm.

The conveyor is the backbone of the assembly process. Its trouble-free operation significantly affects the continuity of production. From time to time however, the bearing of one of the carrier wheels seize and thus the conveyor and, of course, production is stopped. The identification of a given stuck bearing in the wheel and its subsequent replacement often causes a couple of hour’s production outage.

Despite well-planned and implemented preventive maintenance, this anomaly, this problem, occurs at random intervals and causes a problem that must be solved differently than the standard and known approach.

The proposed platform intends to provide an early notification of possible carriers bearing failure, which will allow one to perform preventive maintenance. This prevents assembly line cessation, avoids unwanted downtime in production, and associated financial losses. 

## 2. Literature Review

One of the primary directions of the literature review is relevant for this study. It includes predictive maintenance as a special part of Maintenance 4.0, and also involves approaches related to anomaly detection.

According to [16,17], Maintenance 4.0 involves a holistic view on maintenance as a whole and describes the process focused on data collection, processing in the form of analysis, evaluation, and defines the rules and steps that should be created.

In this document [18], the authors designed the architecture of a predictive maintenance system, based on the Industry 4.0 concept and implemented an analytical tool to predict production equipment failures. In the study [19], the architecture was designed in the context of Maintenance 4.0, taking into account the use of large amounts of data and their subsequent analysis using data mining techniques and methods.

In these articles, the authors focused on the proposal of progressive approaches, which used new methods and technologies, such as the use of cyber-physical systems, artificial intelligence, or supervisory control systems in the architecture design ensuring more efficient implementation of the maintenance process.

The production processes generate a large amount of data. In order to obtain an overview of its behavior, it is necessary to collect, store, and then analyze available data. IoT devices are suitable for collecting process data, as described in [20]. Data collected from any process is possible to use for further analysis. Common statistical methods can be used to analyze the standard behavior of processes. However, if anomalies appear during the process, representing abnormal deviations from the standard state, it is necessary to also use methods and techniques from the field of machine learning, such as support vector machine, artificial neural networks, or k-nearest neighbor. Their use is described by the authors in [21,22,23,24,25]. The results of the performed analyzes can be used for early warning of anomaly [26], classification [27], but also for the reduction of false alarms [28].

In addition to standard behavior, anomalies sometimes occur during manufacturing processes that cannot be clearly identified with standard approaches. To identify them, it is necessary to use methods and techniques of machine learning. Based on the previous cited articles and on the practical experience, it can be stated that the most frequently used approaches include mainly neural networks.

Predictive maintenance according to [29] can be described as a continuous process of verifying the functionality of individual components so as to signal in advance the deterioration of the technical condition of the equipment. Papers [30,31] present an overview of the use of machine learning methods in the field of predictive maintenance and also point to the issue of the correct choice of a particular method.

Article [32] presents the results of the proposed predictive maintenance, using measurements on sensors. The prediction module itself is based on the long short-term memory network and takes into account the requirements of the operators. The authors in [33] focused on building predictive maintenance in the field of railway transport and wind energy, for which they proposed the necessary system requirement specifications, which they then mapped to the capabilities of big data streaming technology.

The survey [34] discusses the individual benefits but also the limitations of predictive maintenance. The authors state that the using of artificial intelligence is increasingly replacing standard approaches. The study in [35] deals with the field of predictive maintenance and draws attention to the challenges related to implementation, but also to the problems associated with it.

In this paper [8], the authors present the possibility of involving intelligent technologies such as smart sensors and smart devices, big data analytics, IoT, augmented reality, and virtual reality.

At present, companies emphasize the continuity of production and reduction of downtime caused by equipment failures. This can be achieved by applying the approaches offered by the Industry 4.0 concept. These include predictive maintenance. The trend of the cited articles clearly leads to the need for approaches that use intelligent devices, and are able to record and monitor the status of production equipment. It is possible to perform analyzes on the obtained data by methods of artificial intelligence and these will enable the identification of the possible non-standard behavior of the devices, which are subsequently eliminated by performing their maintenance.

## 3. Preliminaries

### 3.1. Current State

Our proposal focuses on the predictive maintenance of conveyors on the assembly line in the automotive industry. The assembly conveyer is used to transport car bodies during the entire assembly process. It consists of 59 carriers that move along a closed track with a length of approximately 1250 m. This track is leaded on two height levels, with four carrier’s lifts used to move carriers between them. The track contains places that can be used to temporary decommission carriers for maintenance or due to a failure on the assembly line.

The carrier consists of a transport platform which is used for transporting prefabricated car bodies along a defined railway track. The individual carriers are controlled by their own Programmable Logic Controller (PLC), which cooperates with the company’s Manufacturing Execution System (MES) deployed in the assembly hall. This system provides, among other functions, data on the positions of the individual carriers. The carrier position, however, is not obtained directly from the carrier itself, but is identified only by the scanning gates.

One of the main problems that the company faces during the assembly process is the cessation of the carrier (as a part of conveyor), which can ultimately lead to the cessation of the entire assembly process. For this reason, our proposed predictive maintenance approach aims to reduce production outages caused by problems with carriers.

The most common problems of carrier cessations are caused by the jamming or seizing of a wheel bearing, which carries the entire weight of the transport platform including the car body and workers who perform the assembly process. It should be noted that the transported car body already contains most of the car’s components, such as engine, gearbox, steering subsystem, interior equipment, and so on. The platform is further stabilized by a guide bearing, which keeps it on a railway track, serving to lead it along a specified path in the assembly hall.

The main carrier wheels are connected to the supporting frame of the transport platform using the bearing. The most common problem that occurs is a wheel bearing failure caused by the jammed bearing, due to wheel misalignment, dirt contamination, insufficient lubrication, etc. The consequence of this failure is an increasing bearing temperature to the level of its destruction, which is verified by people from maintenance. Therefore, the temperature increasing can be used as pre-fail parameter to predict the wheel bearing failure. This failure is also accompanied by a whistling sound, which can be heard from a certain frequency in the assembly hall.

The company MES system contains only approximate information about the location of individual carriers, the task of maintenance crew is therefore to visually identify the damaged carrier. After its correct identification, it is necessary to tow the carrier to the nearest service point and replace the damaged bearing, usually together with the wheel. Time required to tow the carrier to the location is given by its distance to the nearest available service location and the time required for the replacement itself. The total time required to eliminate the fault varies from 30 min to 150 min. The consequence of the bearing failure is the cessation of the assembly conveyor behind a given damaged carrier which can lead (and from time to time leads) to a complete cessation of the assembly process.

Other data that could be used for predictive maintenance are not found in the company MES system, given that the PLC focuses only on the control of the carrier’s movement along a predefined path.

For using in predictive maintenance, we decided to measure the temperature of an individual carrier wheel bearings, which should be a sufficient parameter to detect well in advance an emerging problem in the monitored bearing. Providing timely information about a potential bearing problem will give the maintenance crew additional time to inspect the carrier itself.

If the maintenance crew evaluates that the identified problem can cause a wheel bearing jam, they will have enough time to prepare for the replacement and to park the carrier in a suitable position at the service location where the replacement will take place. The bearing and carrier wheel replacement, carried out in this way, can usually take place during the planned break of the assembly line workers, which does not cause the cessation of the entire assembly process.

### 3.2. IoT Sensors Architecture

One of the important prerequisites for the implementation of predictive maintenance was the availability of data from the assembly conveyor line, necessary for the detection of anomalies and evaluation of error conditions.

The collection of temperature data from the assembly carrier, was performed following multiple approaches. To evaluate usability of the Industrial Internet of Things (IIoT) technologies in the selected automotive environment, it was decided to build IIoT infrastructure for the data collection that will future proof the modern production environment [36]. This data collection approach will be further supplemented by stationary temperature measurements, on identified points on the assembly conveyor path.

The design and implementation of IIoT network has focused on the reliability of the network in the assembly hall environment. Therefore, we decided to implement a private IIoT network based on LoRaWAN (Long Range Wide Area Network) technology. This technology was selected after being based on the plant’s recommendations for adopting wireless network technologies.

To achieve sufficient signal coverage on the whole assembly conveyor path, we installed two Kerlink Wirnet Stations [37] operating on the 868 MHz ISM band as our IIoT gateways. These gateways are suitable for operation in an industrial environment and are based on a standard Linux operating system environment and were integrated into the plant’s infrastructure using a common Ethernet connection.

It should be noted that although the area we are covering is a single assembly hall, and the maximum ranges are on the lower end of all used device specifications, two IIoT gateway devices were required to obtain optimal coverage of the whole assembly line and to minimize data loss. This is mainly due to the underground passages that the assembly conveyor is using during travel. Using only one gateway, we detected periodical data losses from the IIoT devices. The insufficient coverage on various spots using only one device was confirmed by the Adeunis ARF8123AA [38]—a field-testing device for LoRaWAN.

For the temperature measurements on the assembly carriers, we utilized LoRaWAN IoT temperature sensors Adeunis ARF8180BA [39], which possess internal as well as external wired temperature sensors. The detailed specification of the used devices can be found in Table 1. These devices were suitable for our deployment, since they provide ambient temperature using the internal sensor as well as the carrier wheel temperature using the external sensor. Due to the continuous assembly line cycle, remote configuration and consumption optimization were required features for these devices.

Although the wheel temperature is a relatively time stable process, the most detailed sampling period of 100 frames per day was selected for the proof-of-concept phase. Using the calculation mode of the device, which sends an average value from the measurements performed over a given period of time, one year of continuous monitoring per battery were expected. Using this configuration, we obtained data from the measurements every 14.4 min.

The specifications of the used temperature sensors, shown in Table 2, were in compliance with the initial requirements for the carrier wheel measurements.

### 3.3. Stationary Sensors Architecture

Data collection from the IIoT network was further supplemented by stationary temperature measurements, on identified points on the assembly conveyor path. As the measurement points were identified, two assembly carrier lifts transfer the carriers from the lower to upper level and carrier after continuous movement. In the identified points is a high probability of carrier wheel temperature increasing during a bearing failure, since the carrier is in continues movement and the bearing did not cool down.

**Figure 1:**
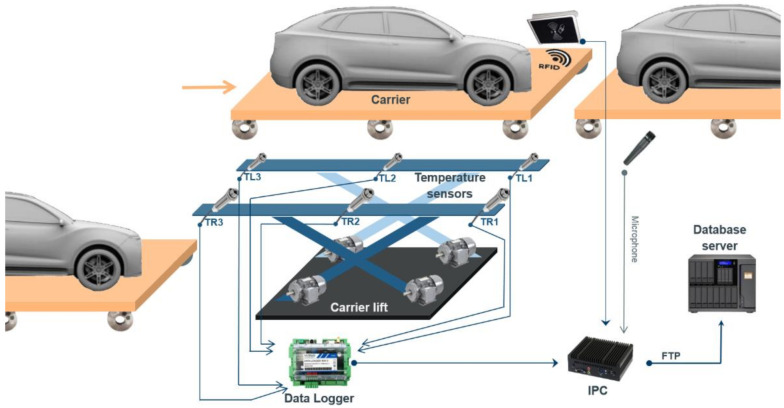
Stationary temperature measurement infrastructure proposal.

One of these measurement points was selected in the proof-of-concept stage to perform the stationary wheel temperature measurements as well as the motor lift parameters, such as lift platform inclination, lift motors current consumption, temperatures, and vibrations, which were also used for predictive maintenance purpose. The proposed measurement infrastructure is shown in Figure 1. It shows the carrier direction of movement as well as the temperature sensors positions and their labels. It should be noted that we will focus solely on the carrier wheel temperature measurements for bearing failure predictions in our paper.

For the data collection from the sensors, we used Viltrus Electronics Data Logger RAY-3 in configuration RAY-3.124.1.0.111333.2.4.10.1 [40]. The selected parameters of this device are shown in Table 3. The main requirements on the device are the support of standard industrial network protocols for communications as well as the ability to perform remote configuration. Since the assembly hall environment is operated and maintained by multiple companies, the galvanically isolated interfaces and power supply proved to be crucial features for long term production deployment.

The 6 analog inputs were used to connect temperature, vibration, and tilt sensors. For the temperature measurements performed on the 6 carrier wheels, data logger devices marked “Ray101” and “Ray102” were used. For the data collection in our implemented application, we utilized the Modbus protocol for direct access to the measured values. After the proper data logger register configuration, this approach worked without major issues.

#### 3.3.1. Temperature Measurement

For the contactless temperature measurements, we wanted to deploy infrared pyrometer sensors from Optris, namely OPTCSTCLT15 [41]. All throughout the sensors were working, however after modifying sensor connections on the data logger we experienced incorrect and unstable measured tension values. The most probable reasons considered were the wrong connections of the Optris sensors or faulty input protection on the data logger device. However, none of these were confirmed.

Since we were not able to fix these issues with the help of Optris support and decided to switch to the Calex PC21MT-0 [42] non-contact temperature sensors, which were used without issues for measurements of temperature on the carrier lift motors. The parameters of these sensors are shown in Table 4. These sensors are suitable for temperature measurement in our environment and provide better accuracy and repeatability than the Optris sensors. Calex also provides air purging collars for dealing with dust that could be added for continuous production deployment.

These sensors were connected using the analog outputs to the data logger devices marked as “Ray101” and “Ray102”. Due to the custom nature of the carrier lift measuring position, custom sensor mounts were created to hold them in place. The measuring distance was around 170 mm for all 6 installed sensors.

The most challenging step in using these sensors, was the proper placement and stability of the sensor mounts to ensure the repeatability of measurements. After the initial installation, the repeatedly measured values were in the ±3 °C range. After the sensor mounts modification, and proper calibration, we achieved an accuracy of ±1.2 °C at the ambient temperature of 26 °C.

#### 3.3.2. Carrier Identification

For carrier identification, Radio-Frequency Identification (RFID) technology was used. The RFID technology was preferred instead of a bar code scanner, due to the poor lightning conditions and continuous setting of impurities on the carrier sides. A RFID reader device was used mid-range SICK RFID RFH630-1000001 [43], its parameters are shown in Table 5. This device was installed near the stationary temperature measurement sensors to provide not only carrier identification but also to identify carrier presence for measurement start signal.

The RFID device was installed on a custom mount, within the 150 mm distance from the carrier side, to achieve error-free tag reading. For the device communication, we used the RS-232 serial interface.

Various types of RFID tags shapes and mount types were tested, however the square shape with the metal base, mounted using industrial glue and secured using translucent adhesive tape, proved to be sufficiently durable.

#### 3.3.3. Industrial PC

For the data collecting and processing on the site, we utilized the Industrial PC (IPC) AAEON NANO-002N-6100-01 [44], whose parameters are shown in Table 6. One of the selection criterions were the availability of the audio inputs and serial port connection. This IPC served as a basis for running our data collection and integration application implemented in Python, and it could be further extended by implementing additional functionalities.

The IPC was supplemented by the Solid State Drive and 60 W power supply from the vendor and was mounted on the measurement site supplemented by the external fan for providing additional air flow. Although the IPC contains two network adapters, only one was used due to the connectivity available on site. The IPC was running the Ubuntu Server 18.04 LTS.

#### 3.3.4. Sound Measurement

For sound measurements, we selected the calibrated measurement micro-phone miniDSP UMIK-1 [45], its specifications are shown in Table 7. This microphone provides manufacturers calibration for frequency response and uses the USB connection compatible with the Advanced Linux Sound Architecture (ALSA) framework in Linux.

### 3.4. Methods for the Bearing Failures Evaluation

For empowering the bearing failure detection in our proposal, we decided to add sound analysis in to the implemented approaches. The monitoring, detection, and diagnostic of bearing faults from sound recordings could be achieved using different methods. Some of them are simple for use while others require sophisticated signal processing.

Overview of methods of the bearings condition monitoring are well summarized in the basic works [46,47,48]. Progress in this field is captured by works [49,50].

They may be generally based on measurements of vibration, acoustic analysis, temperature measurements, and wear analysis. Shocks and bearing faults can be analyzed either in the time domain or in the frequency domain [51].

The time-domain feature extraction method is the most dominant one for rolling element bearings. Scalar descriptors, such as Root Mean Square (RMS), peak amplitude of vibration level, Crest factor, Kurtosis, Skewness [52,53,54], detection of shock waves method, Julien method [55], Cepstrum [56], and multiple other methods are generally used for statistical analysis [57].

The most widely-used frequency domain methods are spectral analysis around bearing defect frequencies [58,59], frequency spectrum in the high frequency domain [60], Spike energy [61,62], high frequency demodulation, acoustic emission focused on high frequency events [63,64,65], adaptive filtering, artificial neural networks, time-frequency, etc.

Acoustics methods are also established as promising for condition monitoring and bearings fault detection [66,67]. Many studies were performed under laboratory conditions with artificially defected bearings instead of bearings with a natural defect. Depending on where the defect occurs, it can excite one of the characteristic bearing fault frequencies [68]:

- Ball Pass Frequency Outer (BPFO),
- Ball Pass Frequency Inner (BPFI),
- Ball Spin Frequency (BSF),
- Fundamental Train Frequency (FTF).

These frequencies, manifested in both vibration and acoustic signals, are detectable with the appropriate processing techniques. The signal of early fault is often too low due to the fact that small localized damage can generate small periodic pulses. Over and above, the measured vibrational signals in the initial stage are disturbed by loud sounds.

## 4. Results

Wheel bearing failure on the roller element makes up a great part of the failures identified on the assembly conveyor carriers. The failure usually results in the wheel bearing seizure, blocking the movement of the carrier and resulting in the temporary shutdown of the conveyor. Therefore, the detection of the wheel bearing failure through any monitoring process is especially important.

Online condition monitoring, proposed in our paper, is therefore highly desirable due to the potential issues that can be detected and rectified during scheduled maintenance. The proposed and implemented solution incorporates requirements for the design and implementation of the monitoring system, which will provide early warning of potential failures of the carrier wheel bearings, in order to minimize the occurrences of unexpected failures.

**Figure 2:**
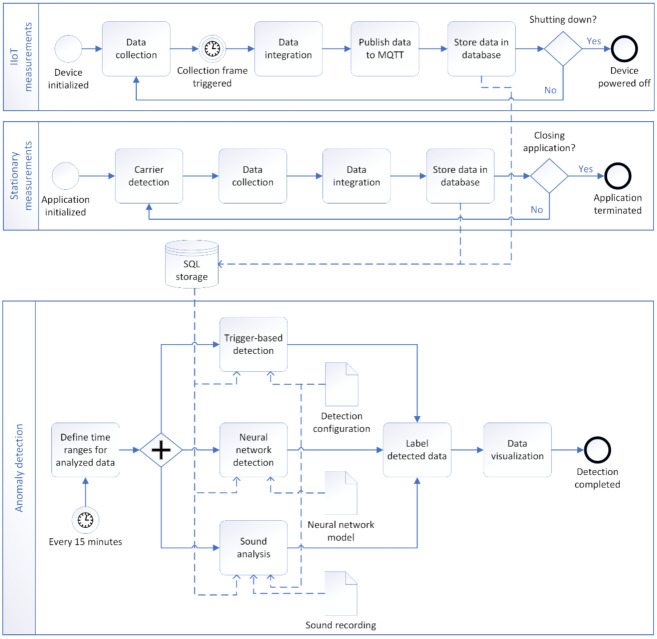
Process diagram of proposed monitoring and anomaly detection system.

The proposed system covers multiple processes, involving data collection and their processing, data storage, and anomaly detection process, as shown in the process diagram shown in Figure 2.

The diagram outlines the processes required for the monitoring and anomaly detection of the wheel bearing failure proposed in our paper. Data collection using the IIoT devices is performed by the IIoT devices alone as is further described in Section 4.1. The stationary measurements performed in our proof of concept are carried out, implementing the algorithm described in detail in Section 4.2.

The anomaly detection approach consists of following methods that were proposed and implemented in our solution:

- Trigger-based bearing failure detection,
- Anomaly detection using a neural network,
- Bearing condition detection using sound measurements.

These methods can operate separately and are combined in our solution to provide a comprehensive overview of the bearing wheel status, detected anomalies, and bearing failures themselves. Used methods are described in detail in Section 4.3, Section 4.4, and Section 4.5.

All through the implemented monitoring and detection system is fully automated, the results evaluation is in the hands of the maintenance crew, since the maintenance and repair scheduling as well as the detected anomalies evaluation is carried out by them.

### 4.1. Data Collection and Integration Architecture for IIoT Measurements

For all the installed sensors and devices, it was necessary to design a data collection and integration process to obtain suitable data for further analysis. Since we utilized two data collection technologies, this approach can be divided into two separate designs that both integrate their data into the common data storage. As the main data storage, we utilized Yandex ClickHouse column-oriented database that can be used for efficiently storing and accessing time-series data.

**Figure 3:**
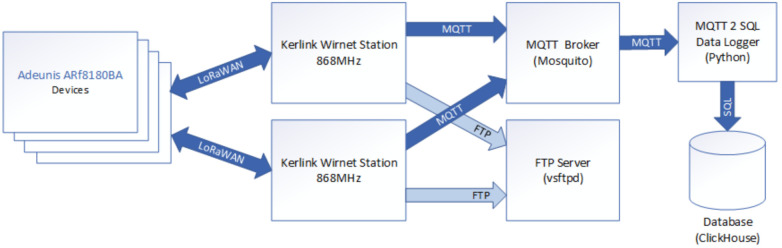
Designed and implemented data collection and integration architecture for Industrial Internet of Things (IIoT) measurements.

For the collection of data originating from the LoRaWAN devices, we designed and implemented the architecture shown in Figure 3.

The data measured on the IIoT devices are transferred through the LoRaWAN network to the available Kerlink Wirnet Station in range. Since we are using multiple stations with overlapping coverage, receptions of the same data on both stations are quite common. Deduplication of received data was therefore required and performed in data collection application implemented in Python.

To achieve near real time data storing, we utilized the Message Queuing Telemetry Transport (MQTT) protocol that is provided by the Kerlink Wirnet Station. The MQTT broker was utilized in the middle for data buffering and future expansion possibilities. In our proof of concept, we utilized the lightweight Mosquitto MQTT broker that met all requirements.

For the transfer of MQTT data into the database server, a custom Python application using paho-mqtt library was created. This application was subscribed into the MQTT broker topics and transformed the obtained message payloads into the measured values.

**Figure 4:**
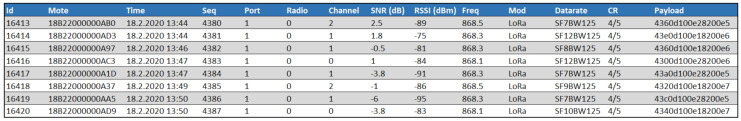
Sample of IIoT data collected from the Adeunis devices.

Sample data from the Adeunis devices, received by the Kerlink gateway, are shown in Figure 4. These data contain the necessary device identification, defined as a Mote number, Time value, and Payload containing RAW binary data. This binary data had to be further transformed into measured values.

**Figure 5:**
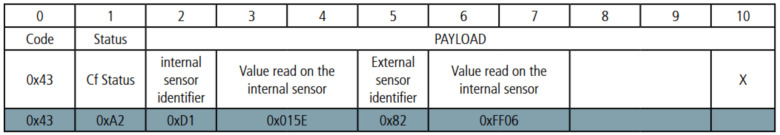
Adeunis device payload structure.

To decode the Payload value, official DataFrame documentation of the Adeunis devices was used. The device payload structure, shown in Figure 5, served as a basis for the decoding process. The values of internal and external sensors are stored in the Big Endian byte order and therefore had to be transformed to obtain measured values. Due to the fact that both sensors were part of the same device, the values could be transformed directly without any need for further calculation.

This conversion was supplemented by decoding the Status bits, to identify error states and low battery warnings, since the identification LEDs on the devices are not accessible during use and the devices do not provide any other status information.

For the data archiving and debugging purposes, all the data received from both LoRaWAN gateways are stored on the File Transfer Protocol (FTP) server, using the options provided by the Kerlink Wirnet Stations. This process is performed periodically every 60 min. The target FTP server was installed on a CentOS server in the plant’s infrastructure.

### 4.2. Data Collection and Integration Architecture for Stationary Measurements

The data obtained from the stationary measurements shared the data collection infrastructure with the carrier lift sensors for the lift platform inclination, lift motors current consumption, temperatures, and vibrations. However, in our paper, we solely focus on the carrier wheels temperature measurement.

**Figure 6:**
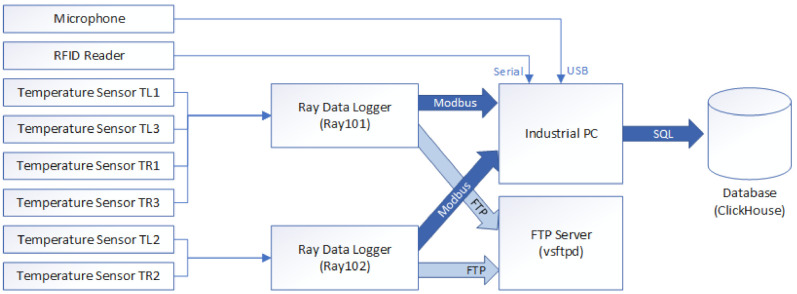
Designed and implemented data collection and integration architecture for stationary measurements.

Since we decided to incorporate the sound recording for the carrier bearing failure identification, we installed the measurement equipment near the stationary temperature measurement. The data collection and integration architecture for the carrier wheels temperature measurement and carrier sound recording is shown in Figure 6.

Due to the shared infrastructure, temperatures of the front and rear carrier wheels are collected by the Ray Data Logger marked as Ray101 and middle carrier wheels are collected by the device marked as Ray102. The respective analog inputs on the Ray Data Logger device were properly configured for the input types and ranges of the used temperature sensors.

For the individual analog input values, accessed through the Modbus protocol, was also required to properly set the Ray Data Logger registers. It should be noted that although the Ray Data Logger can perform basic input value transformation, the obtained Modbus values are always in raw input format.

For the transformation of the measured current value into the measured temperature for the Calex sensor, we used:

(1) $$T=I_{\mathrm{out}}*\;\frac{\mathrm{MaxValue}}{20\;\mathrm{mA}-4\;\mathrm{mA}}$$

where T is the measured temperature, I_out_ is the measured current on sensor output, MaxValue is the maximum measurement range of the sensor, 20 mA is the largest output current of the sensor, and 4 mA is the lowest output current of the sensor.

The RFID reader device is connected to the Industrial PC using the standard RS-232 connection. The measurement microphone is connected directly into the USB port on the IPC.

The IPC runs the data integration application, implemented in Python programming language, for collecting and integrating the measured data. For the data reading in designed application, we used the PyModbus library for Modbus communication, PySerial library for serial communications, and PyAudio library for sound recording.

For data backup and debugging purposes, we utilized the data logger function to store the data every 30 min on the FTP server. This server was shared with the IIoT gateway backups.

**Figure 7:**
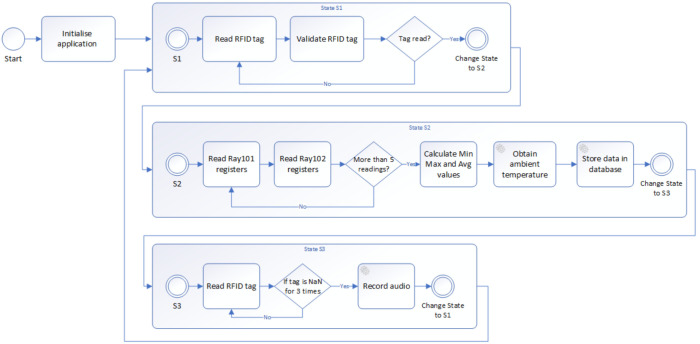
Designed measurement algorithm.

For the stationary measurement of the carrier wheels temperature on the carrier lift, a custom measurement algorithm was designed, as shown in Figure 7. The measurement of the carrier wheel temperatures has to be performed during and after the carrier lift up process that is independent from the temperature measurement.

The measurement algorithm is designed around three stages that are looped indefinitely. The first state focuses on the RFID tag reading, since the valid tag is the signal for starting the carrier wheels temperature measurement. The RFID reader uses a serial bus with a 9600 baud rate. Since the RFID reader uses a serial bus for communication, the RFID tags are validated against the tag pattern and also against the RFID tag list that contains all installed RFID tags paired to the carrier ID number. When the tag is read and validated, the program is switched to the second state.

In the second state, the program reads the data from the Ray Data Logger registers and converts them into the measured values, based on the type of sensor. For the used temperature sensors, the value is converted using Equation (1). The temperature is measured every second for five times, to increase the accuracy of the measurements. When the measurements are completed, the measured values are used to calculate minimal, maximal, and average values and are subsequently stored in the database. It should be noted that the saving of data into the database is performed asynchronously, to minimize the unpredictable events influence on the application logic. Finally, the algorithm is switched to the third state.

In the third state, the measurement algorithm waits until the carrier starts to move away from the lift. The RFID tag reading must fail three times before we declare the carrier as moved. This is due to the tag reading issues despite the fact that the carrier RFID tag was still present. When the carrier has moved, we start the sound recording that is performed asynchronously in the separate application thread.

**Figure A1:**
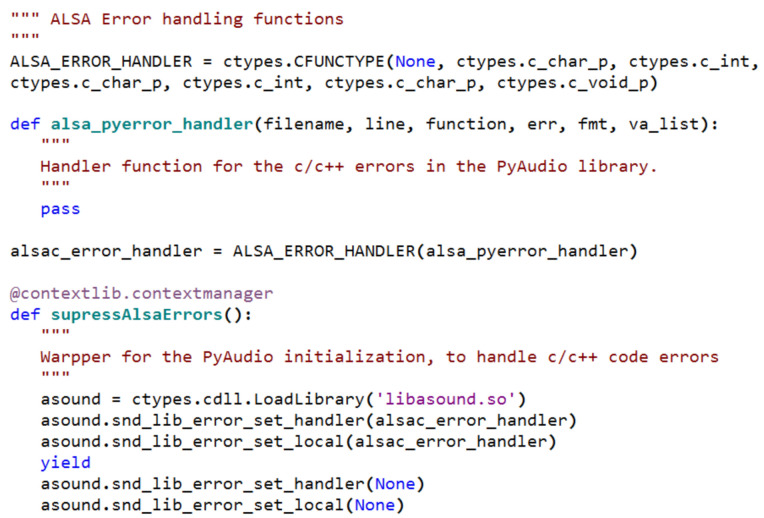
Python code for custom ALSA error handler.

The audio sample recorded for each carrier has 58 s and is recorded as a mono channel with a 16-bit word size and sampling rate of 48 kHz. For the recording in Python, we used the PyAudio library that provides the wrapper over the standard Linux ALSA library. One of the biggest issues of the PyAudio library was the underlying ALSA library written in C programming language. During continuous deployment, multiple issues arose caused by the C errors not captured and handled properly by the PyAudio library. Therefore, we had to implement a custom ALSA error handler, shown in Figure A1 in Appendix A.

It should be noted that we had omitted the argument parsing using the libc Python library and regular expression error matching in the code sample, for better clearness and simplicity. This code is usually recommended for ALSA device initialization, in our proof of concept we found that it is also necessary for audio stream recording. This was due to the occurrences of random ALSA errors during the recording. These errors occurred randomly, without any configuration or library version changes in the system.

The recorded audio samples were stored on the FTP server, shared with the Kerlink, and RAY-3 data backups. One recorded sample has an average size of 5 MB and we kept all recorded samples stored for further audio analyses. To save the space, audio files older than one month were automatically deleted using scheduled Bash script.

### 4.3. Bearing Failure Detection Using Trigger Based Approach

Due to the fact that the IIoT temperature measurements were implemented as first in our proof of concept, we could use this data for further analyzes. From this data we removed bearing failure records and assembly downtime during the weekends, to represented the carrier wheel temperature values measured during normal operating conditions.

For the trigger-based bearing failure detection, using the carrier wheel temperature measurements, we decided to use common approach by using the wheel temperature threshold. The carrier wheel temperature was measured using the external contact temperature sensor on the Adeunis IIoT device.

**Figure 8:**
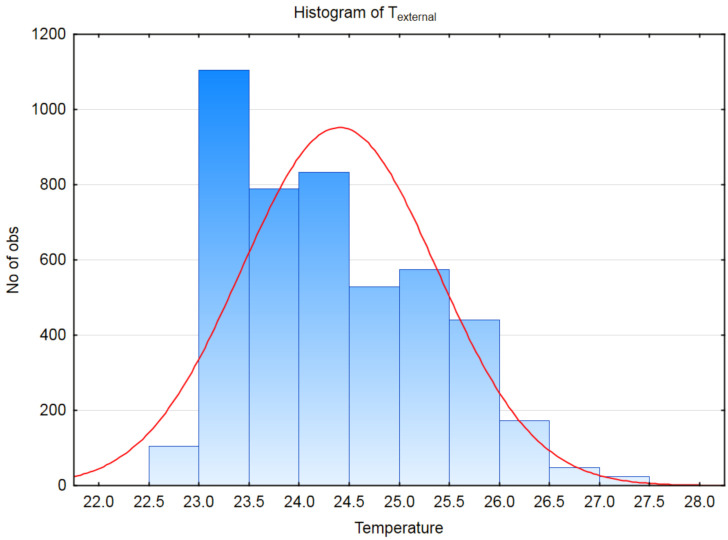
Histogram of T_external_ parameter.

From the IIoT data obtained during a period of two weeks collected in February 2020, we created a histogram, shown in Figure 8. The histogram illustrates the number of observations of measured carrier wheel temperatures from the IIoT devices. It can be seen that the majority of measured temperatures start at 23 °C and end at 26.5 °C, with the average temperature at around 24.5 °C. This clearly shows that the carrier wheel temperature during the operational conditions do not contain outliers that could affect the bearing failure detection using the carrier temperature measurement, since the carrier wheel temperature during the bearing failure is significantly higher.

To compensate the data errors in the early stage of the proof of concept, we collected minimum, maximum, and average wheel bearing temperature values. In the proposal we decided to use the averages of measured carrier wheel temperatures, provided by the IIoT and stationary devices.

For detecting the carrier wheel temperature anomalies for predicting the bearing failure, we considered multiple approaches. These include for example:

- Fixed temperature difference;
- Difference against the average wheel temperatures of the carrier;
- Difference against the wheel temperatures on previous carriers;
- Difference against ambient temperature.

We evaluated proposed trigger-based detection approaches and performed tests using the collected data to select the most suitable for detecting wheel temperature anomalies. Although the fixed temperature threshold detection method worked, it required manually changing the detection value due to the unstable temperature in the assembly hall. Evaluating the difference against the wheel temperature on other carriers seemed promising, however this approach generated a large number of false positive or false negative detections before and after daily breaks in production. This was caused by the stationary carriers, i.e., the carrier wheels cooled down during the daily break.

Therefore, we selected the difference against the average temperature of other carrier wheels and difference against the ambient temperature for trigger-based detection implementation. The detection parameters were specified based on the statistical analyses of collected data. These parameters were subsequently tweaked using following months data.

The selected approach was also further supplemented by the data from the stationary temperature measurements. The correlation matrix in Table 8 clearly shows strong correlation between the ambient temperature (TAmb) in the assembly hall and temperatures measured on carrier wheels. Measured temperatures are labeled as TR1—TR3/TL1—TL3 for the wheel temperature on the right/left side of carrier, numbered from the front side of the carrier. There is also a strong dependence between the wheels on the opposite sides of the carrier. It should be noted that the correlation matrix was created on the data without carrier wheel failures, to show normal operation temperature dependencies.

**Figure A2:**
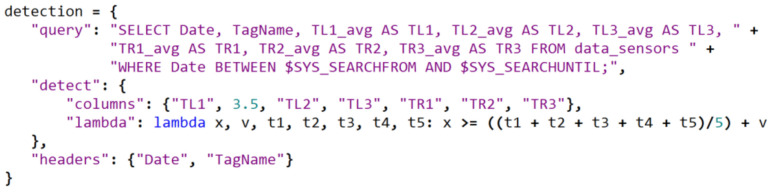
Python code for trigger-based anomaly detection.

To implement the trigger-based carrier wheel temperature anomaly detection for predictive maintenance, we proposed an algorithm for evaluating data stored in the database and notifying assembly hall maintenance. The algorithm was designed to provide possibilities for detecting and evaluating any of the proposed methods. This was done by using the lambda expression for trigger function definition, as shown in the detection configuration code in Figure A2 in Appendix A. Detection configuration uses the difference against the average wheel temperatures of the carrier.

The query parameter specifies the query used for obtaining the data from the database for the trigger function. The detect dictionary structure specifies the table columns used for the detection, and the lambda function that is evaluated. If the lambda function is evaluated as true, the detection was successful. The headers item defines array of database columns that will be added to the detection record for further processing.

This algorithm was subsequently implemented in the application, developed in Python programming language and performed scheduled data evaluation every 15 min. The detected anomalies in the data were labeled and further processed by email and SMS notification functions to notify assembly hall maintenance.

**Figure 9:**
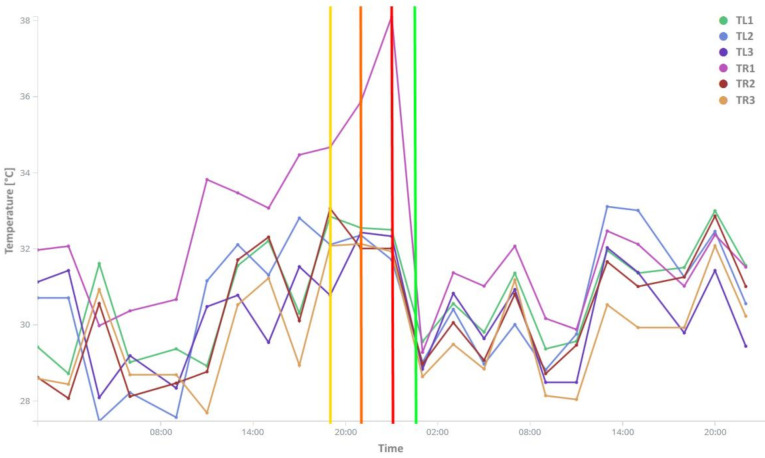
Carrier bearing temperature before and after predictive maintenance.

The results of the implemented trigger-based detection approach can be clearly seen in Figure 9, which shows the bearing failure on carrier 25 identified using the temperature anomaly detection approach. The line graph shows the temperature profile of all carrier 25 wheels before and after bearing failure.

The bearing failure on wheel TR1, in this example, was detected at 19:34 (yellow line in Figure 9) using the difference against ambient temperature. This detection was further supplemented by the difference against the average wheel temperatures of the carrier detection approach at 21:42 (orange line in Figure 9). The anomaly was further confirmed at 23:43 (red line in Figure 9) by both detection methods and the maintenance crew was notified. The bearing wheel failure was then repaired around 00:40 (green line in Figure 9) at the next carrier rotation and was returned back into the assembly conveyor without any unplanned production downtime.

The detection sensitivity of the used methods is therefore sufficient for the bearing failure detection, and allows carrier bearing repair to be performed well in advance by the maintenance crew.

### 4.4. Neural Network Design for Bearing Failure Detection

When we want to further move detection time before failure occurrence, the main limitation of the trigger-based anomaly detection approach is increasing false detections. To improve this, we decided to use machine learning approach. From published articles [23,24,25] and previous projects [69,70,71], the neural network was evaluated as suitable for this use due to the excellent results with the numerical values, its possibility to improve results based on future data, and suitability of implementation for our smart system.

After collecting a sufficient amount of data, which was continuously collected during the initial phase of proof of concept, it was possible to begin with the neural network design, which will be used to predict the failure conditions of the carriers’ wheel bearings. The initial phase started in March 2020 with the stationary measurement infrastructure installation, however the data collection started in September 2020 after measurement infrastructure testing and acceptation.

For the neural network design, we used the data science platform TIBCO Statistica, which provides various tools not only for the analysis and preprocessing of the input data set, but also for the design and subsequent optimization of the neural networks.

The first step was to analyze the available data set. During the six weeks of proof-of-concept operation, approximately 16,000 data records were collected from the carriers. Temperature was measured on all six carrier wheels and stored in the ClickHouse database. This data set was supplemented by the temperature measured in the assembly hall, which affects the temperature of the carrier wheel bearings. During the proof-of-concept operation, 27 failures occurred on the carriers, and 18 of them were caused by a fault in the wheel bearings.

The bearing temperature data set is necessary to be enriched by the bearing repair information from the maintenance logs, which contained the date and time of the repair, and type of the failure occurred. This information was added into the data set manually using the information from to the maintenance crew and is available in their maintenance log. This information was later used in the neural networks training processes for the carrier bearing failure prediction. It should be noted that we excluded repairs that were not related to the wheel bearings.

Subsequently, it was necessary to determine whether the data set did not contain values significantly different from other values, usually referred to as outliers. It was important not only to determine whether the data set contained such values, but also the reason for the existence of such values. The different values could be caused by e.g., reading error from the temperature sensors, its incorrect transformation, or whether it could have been the correct values. The input data set contained some values with significantly higher temperatures, but these values were marked by maintenance personnel as values that led to bearing failure. The data set was further examined for missing or noisy data in its parameters caused, for example, by unsuccessful value reading from the temperature sensors or by a connection failure during the database operation, but such data did not appear in the input data set.

For the designed neural network, we set the Bearing Failure parameter as the target attribute for prediction of the bearing failure on the carrier wheel. This parameter evaluates whenever the bearing was evaluated as failing and repaired by the maintenance crew, which was obtained from the maintenance logs. Since the wheel bearing temperature increases before the failure is continues, this parameter, represented by the Boolean value was set for four measured temperature values before the failure on the identified carrier. The bearing failure was represented by Failure (True) value and the normal operation was represented by OK (False) value.

**Figure 10:**
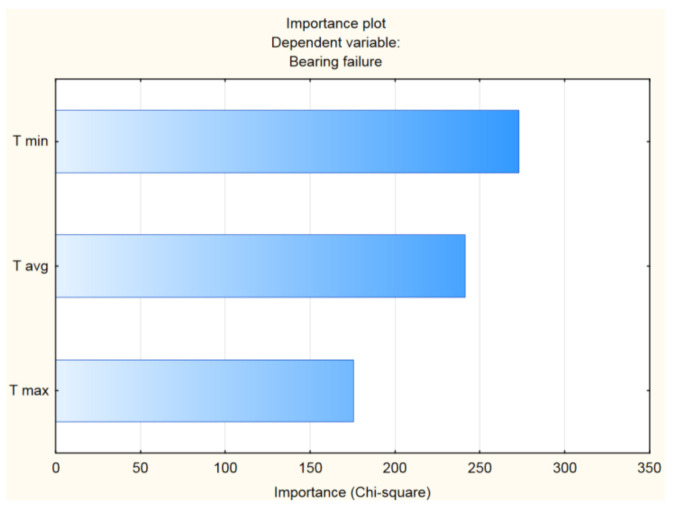
Importance plot of “Bearing failure” parameter.

Since there are storing minimum, maximum, and average temperature values from each carrier wheel measurement we had to evaluate which of these parameters group was more suitable for neural network inputs. The dependencies in the data set were analyzed using importance plot analysis, shown in Figure 10. This allow us to reduce the number of input parameters that need to be used in the neural network design.

Based on this result, we decided to use the minimal and average temperature values for each carrier wheel as input parameters for our neural network. These parameters were supplemented by the ambient temperature measured in the assembly hall, since the wheel temperature is directly dependent on the ambient temperature, as shown in Table 8. All neural network input parameters are represented as decimal numbers.

It should be noted that the number of bearing failures that occurred during data collection was quite small in relation to the total amount of data records. Due this fact we decided to create data subset using Stratified sampling, which would mitigate the impact of data imbalances on bearing failure prediction [72,73,74].

The input data set was divided into training, testing, and validating sets in the ratio of 60%—20%—20%. Using the Automated network search function, provided by the TIBCO Statistica data science platform, several neural networks for the predicting of the bearing failure were designed. The obtained MultiLayer Perceptron (MLP) neural networks differed in various numbers of neurons in the hidden layer, type of activation and error functions, used training algorithm etc. Based on achieved performance in all subsets and their balanced variance, the most suitable neural networks are shown in Table 9.

For the suitable neural networks, we created confusion matrixes and calculated selected performance evaluations parameters. To empower the validation data set we had to decide to supplement it with data from the following five weeks during which 16 bearing failures occurred. This resulted into 87 carrier wheel temperature measurements to be labeled as Failure.

From the performance evaluation parameters, we calculated the neural network precision, recall, accuracy, and *F_1_ Score* representing harmonic mean of the recall rate and precision as:

(2) $$\mathrm{Precision}=\frac{\mathrm{True}\;\mathrm{positives}}{\mathrm{True}\;\mathrm{positives}+\mathrm{False}\;\mathrm{positives}}$$

(3) $$\mathrm{Recall}=\frac{\mathrm{True}\;\mathrm{positives}}{\mathrm{True}\;\mathrm{positives}+\mathrm{False}\;\mathrm{negatives}}$$

(4) $$\mathrm{Accuracy}=\frac{\mathrm{True}\;\mathrm{positives}+\mathrm{True}\;\mathrm{negatives}}{\mathrm{True}\;\mathrm{positives}+\mathrm{False}\;\mathrm{positives}+\mathrm{True}\;\mathrm{negatives}+\mathrm{False}\;\mathrm{negatives}}$$

(5) $$F_{1}\;\mathrm{Score}=2*\;\frac{\mathrm{Precision}*\mathrm{Recall}}{\mathrm{Precision}+\mathrm{Recall}}$$

where *True positives*, *False positives*, *True negatives*, and *False negatives* are calculated from the neural network prediction spreadsheet.

After the parameter evaluation, the neural network labeled MLP 13-18-2 with 13 neurons in the input layer, 18 neurons in the hidden layer, and 2 neurons in the output layer, was selected as most suitable. The confusion matrix of this neural network is shown in Table 10.

The selected performance evaluation parameters of the most suitable neural network are shown in Table 11.

The selected neural network is able to predict the bearings failure of the carrier based on the temperature of wheel bearings and the temperature in the assembly hall. During several tests with different input data sets, a neural network was selected capable of predicting the failure state of the carrier wheel bearing on average 4 to 6 h before the probable failures. Based on these evaluations, the selected neural network was accepted as a suitable to integrate it into the designed application.

For neural network implementation, we utilized TIBCO Statistica data science platform which provides the ability to generate the neural network source code. This code was subsequently integrated into the implemented application written in Python.

### 4.5. Sound Measurement System and Analyses Procedure for the Bearing Condition Detection

According to the experience of the maintenance department, the gradual seizure of the bearings is always accompanied by acoustic noise in the form of whistling. This was also confirmed during the regular inspections of the function parts condition of the carrier and assembly conveyor.

By analysis of sound recordings, in the time and frequency domain, it looks for characteristic acoustic manifestations of bearing damage. This approach of early prediction does not allow identify specific bearing on which damage occurs. Therefore, this condition monitoring system works in the cooperation with the subsystem for measuring the temperature of individual bearings.

An acoustic records of bearing failure, used as an example in this paper, were obtained from real operation on two carriers marked 25 and 44. These records were evaluated by the time domain statistical indicators and frequency analysis.

**Figure 11:**
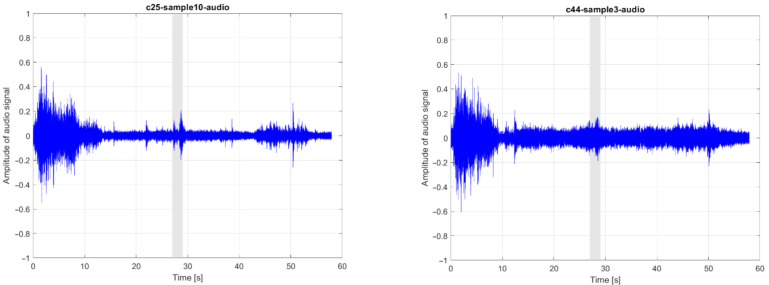
Recorded audio tracks with a highlighted area of whistling sound from: (**a**) Carrier 25 and (**b**) carrier 44.

When any bearing was damaged, a whistling sound was generated randomly at various carrier positions on the conveyor. However, the whistling always occurred when the carrier moved on the conveyor rail. All audio recordings were made at the same position on the assembly conveyor. The whistling sound is always recorded in them between 27 and 29 s, as highlighted in Figure 11a. To confirm this finding, Figure 11b presents the sound record obtained from the carrier 44 with a faulty bearing. This eliminates the problem of how to search the whistle in the long-time record and reduces the likelihood of accidental whistling in the background. For carrier marked 25, 14 audio records were stored, one of them after bearing replacement. The audio tracks were recorded approximately two hours apart.

#### 4.5.1. Analysis of Acoustic Signal in Time Domain

In time domain statistical scalar indicators which assess the signal amplitude are used for bearing diagnosis. As is known from the literature [53,75], standard scalar indicators (RMS, Kurtosis, Crest factor, and Skewness) are less sensitive when the number of defects increases. Due this reason, the audio signal was also assessed by applying the Scalar factor, Impulse factor, and new TALAF and THIKAT indicators defined in [76]. These new indicators combine values of standard scalar indicators into one unit. The selected commonly used statistical descriptors, used in time domain analyses, were calculated using equations:

Peak

(6) $$\mathrm{PK}={\mathrm{Sup}}_{1\leq i\leq N}\left|x_{i}\right|$$

Root mean square

(7) $$\mathrm{RMS}=\sqrt{\frac{1}{N}\overset{N}{\underset{i=1}{\sum }}x_{i}^{2}}$$

Crest factor

(8) $$\mathrm{CF}=\frac{\mathrm{PK}}{\mathrm{RMS}}$$

Kurtosis

(9) $$\mathrm{KURT}=\frac{\frac{1}{N}\sum_{i=1}^{N}{\left(x_{i}-\bar{x}\right)}^{4}}{{\mathrm{RMS}}^{4}}$$

Skewness

(10) $$\mathrm{SKW}=\frac{1}{N}\overset{N}{\underset{i=1}{\sum }}{\left(\frac{x_{i}-\bar{x}}{\mathrm{RMS}}\right)}^{3}$$

TALAF

(11) $$\mathrm{TALAF}=\log\left[\mathrm{KU}+\frac{\mathrm{RMS}}{{\mathrm{RMS}}_{0}}\right]$$

THIKAT

(12) $$\mathrm{THIKAT}=\log\left[{\mathrm{KU}}^{\mathrm{CF}}+{\left(\frac{\mathrm{RMS}}{{\mathrm{RMS}}_{0}}\right)}^{\mathrm{PK}}\right]$$

Shape factor

(13) $$\mathrm{SF}=\frac{\mathrm{RMS}}{\frac{1}{N}\sum_{i=1}^{N}\left|x_{i}\right|}$$

Impulse factor

(14) $$\mathrm{IF}=\frac{\mathrm{PK}}{\frac{1}{N}\sum_{i=1}^{N}\left|x_{i}\right|}$$

where $\bar{x}$ is the average value of signal samples x_i_ in considered time window, N is number of samples, and RMS_0_ is the root mean square value defined for a healthy bearing. After replacing of the damaged bearing with a new one, the first calculated value of RMS has been used as the RMS_0_ value in our solution.

**Figure 12:**
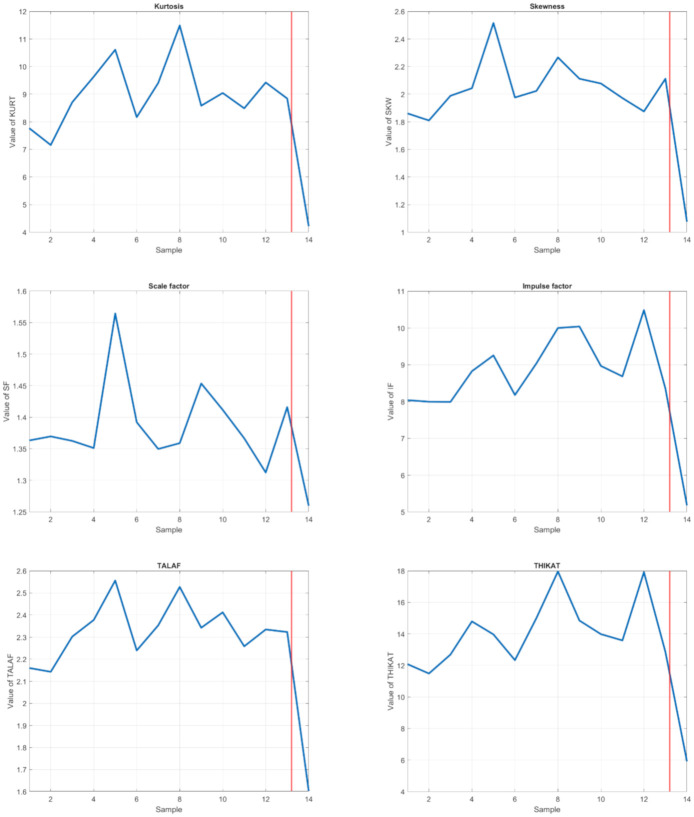
Evolution of the meaningful statistical descriptors for carrier 25 (the red line in the graphs indicates the time of replacement of the damaged bearing).

The statistical descriptors, described above, were considered for analysis of acoustic signal parts of interest during which the whistle sound has occurred. Graphs in Figure 12 shows the evolution of the Kurtosis, Skewness, SF, IF, TALAF, and THIKAT indicators according to the samples ordered by time on the horizontal axis.

Generally, the conventional scalar indicators, such as the Peak and RMS, increase with smaller number of defects. When the bearing damage is moderate, the Kurtosis is the most sensitive indicator of the signal energy density. With the deterioration of the defects, the Impulse factor becomes the most sensitive indicator. The Shape factor is insensitive to damage spread. The TALAF parameter and THIKAT parameter describes in detail the evolution of the damage and achieves bigger difference between faulty bearing and bearing without failure. All observed statistical indicators decreased more or less significantly after the replacement of the damaged bearing with a new one.

The changes of the scalar indicators of the damaged bearing on carrier 25 are presented in the line graphs in Figure 12. The largest value difference between damaged bearing and new one shows the Kurtosis (63.3%) and the smallest difference is in value of the SF (13.1%) indicator. The THIKAT displayed the highest difference between its values for the damaged and new bearing over 67%. The TALAF indicator decreased by 37.5% after bearing replacement. The last two scalar descriptors, Skewness and *IF* reduced after the bearing repair their size to about half the previous value.

Due to these reasons, THIKAT and Kurtosis were selected as the assessing criteria of bearing state for time domain analysis in the proposed condition monitoring system.

#### 4.5.2. Analysis of Acoustic Signal in Frequency Domain

Due to the possible influence of the time analysis results by the assembly hall background noise from other sites near conveyor, the sound record of the carrier passage through the measurement place was also analyzed in the frequency domain.

The fast Fourier Transform (FFT) transformation was utilized for digitalized audio signal analyzes in the frequency domain. The Hanning window function was applied on data blocks to zero-valued outside of the chosen interval and controlling leakage. The sample overlapping was not applied.

One of the main symptoms of the bearing defect are ball-pass frequencies in the frequency spectrum. However, it is usually difficult to locate these frequencies in the spectrum if they are hidden in the background noise. Bearing defect characteristic frequencies are therefore calculated using following equations:

Ball-passing frequency of the outer race

(15) $$\mathrm{BPFO}=\frac{N}{2}f_{r}\left(1-\frac{\mathrm{BD}}{\mathrm{PD}}\cos\alpha \right)$$

Ball-passing frequency of the inner race

(16) $$\mathrm{BPFI}=\frac{N}{2}f_{r}\left(1+\frac{\mathrm{BD}}{\mathrm{PD}}\cos\alpha \right)$$

Ball-spin frequency (about its axis)

(17) $$\mathrm{BSF}=\frac{\mathrm{BD}}{\mathrm{PD}}f_{r}\left[1-{\left(\frac{\mathrm{BD}}{\mathrm{PD}}\cos\alpha \right)}^{2}\right]$$

Fundamental train frequency

(18) $$\mathrm{FTF}=\frac{f_{r}}{2}\left(1-\frac{\mathrm{BD}}{\mathrm{PD}}\cos\alpha \right)$$

where N is the number of rolling elements, f_r_ is the relative frequency between the outer and inner race, BD is the rolling element diameter, PD is the pitch circle diameter, and α is the contact angle. The bearing frequencies of the spherical roller bearing type “SKF 22206 E”, for different speeds of the carrier, are calculated using Equations (15)–(18) and summarized in Table 12. It should be noted that the carrier speed varies in different operating modes. In standard operating mode its speed is approximately 10 m/min. The diameter of our carrier wheel is 140 mm.

Where the parameter *v* is the speed of the carrier and f_rp_ is the over-rolling frequency of the damaged point on the rolling element (roller defect frequency).

**Figure 13:**
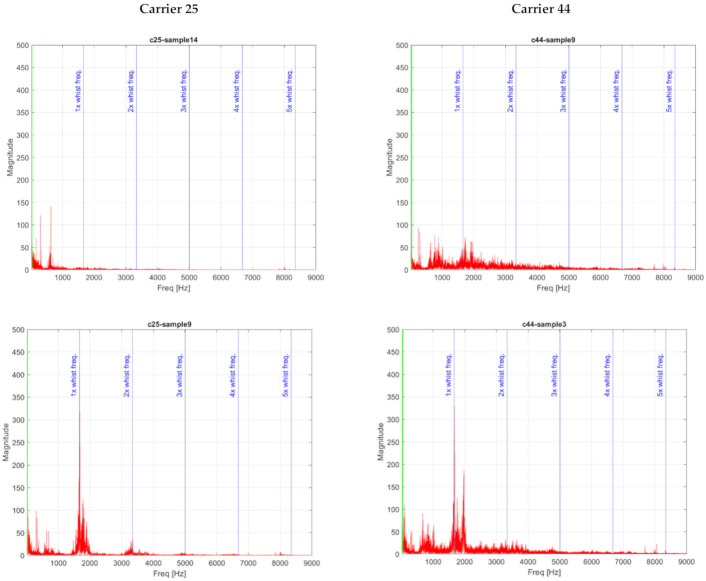
Typical composition of frequency spectrum of the healthy (**top**) and fault bearings (**bottom**) for carriers 25 (**left**) and 44 (**right**).

The typical frequency spectrum of audio signals, recorded at a measurement point for selected carriers 25 and 44, are shown in Figure 13. Graphs in the second row in Figure 13 show examples of fault wheel bearings frequency spectrum. The spectra in the first row correspond to a new bearing after the replacement. The spectra of fault bearings show a large increase in amplitude at the whistling frequency of approximately 1670 Hz and a small rise in harmonic frequencies. Together with this whistling frequency, there is also another significant frequency in the spectrum that is approximately 300 Hz higher. The cause of the whistling is the friction of the steel transport or guide wheel with a seized bearing. The presence of these frequency components in the frequency spectrum indicates whistling and can predict possible bearing damage on the carrier.

Identification of the bearing frequencies in the frequency spectrum of an acoustic signal is a very difficult due to small values being at the limit of FFT resolution. Due to the low carrier speed are the bearing frequencies (BPFO) localized in the range of lower microphone sensitivity, it is difficult to identify them in the frequency spectra. In Figure 13 these frequencies are highlighted by a green line. Based on the analysis of the acoustic signal of a healthy and damaged bearing, it can be stated that the damaged bearing produces an amplitude increase in the frequency spectrum at the 1670 Hz frequency, which corresponds to the whistling generated when the wheel with damaged bearing comes into contact with the carrier guide line.

**Figure 14:**
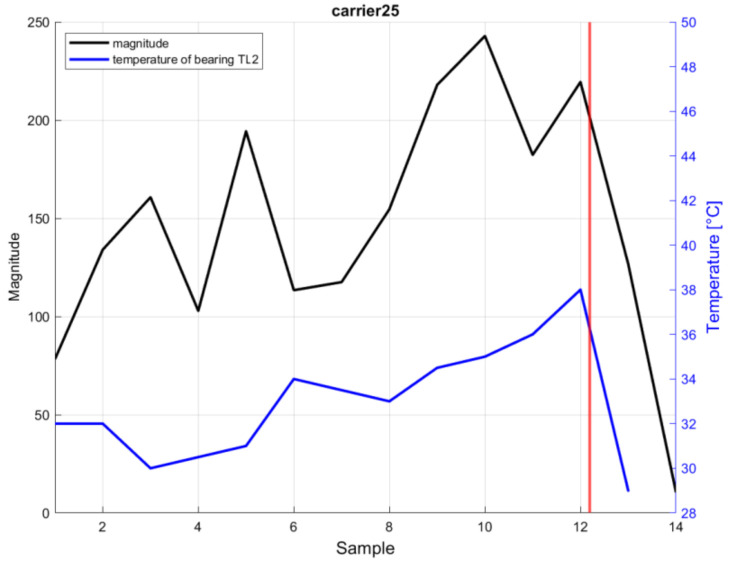
Evolution of magnitude at frequency 1670 Hz in individual samples for carrier 25 and comparison with temperature trend on defective bearing TL2 (the red line in the graphs indicates the replacement time of the dam-aged bearing).

Figure 14 shows the trend of increasing magnitude at frequency 1670 Hz, depending on the order of samples in time. The results of the analysis show a significant decrease in the amplitude of the whistling frequency after the replacement of the damaged bearing for both analyzed carriers. Figure 14 compares the trends of the amplitude increased at the characteristic frequency and the temperature of the damaged bearing on carrier 25.

The proposed algorithm for early condition monitoring of bearings state is based on the analyses of recorded sound signals in the time domain by calculating the selected descriptors. Using the frequency analysis, we detected the presence of magnitude increase for frequencies in range ±20 Hz around the whistle frequency. To identify the damaged bearing, the results of individual bearings temperature measurements have to be applied.

Descriptors Kurtosis and THIKAT were used as auxiliary indicators to assess the condition of bearings by analyzing the audio recordings in the time domain. The magnitude value is tested in the frequency spectrum at a critical frequency of 1670 Hz. When the value of the Kurtosis indicator exceeds 5, the THIKAT value reaches 7 and the magnitude of the frequencies in the critical frequency range is greater than 100, the carrier is included in the group with a potentially damaged bearing. This can be further investigated by the maintenance personal to analyze the bearing state for future assembly conveyor modifications. This identification was also used to minimalize anomalies, which occurred in the implemented trigger-based bearing failure detection, using the carrier wheel temperature measurements.

In our proof-of-concept phase, acoustic treating of the sound recording point was not possible and an alternative had to be found. Therefore, the magnitude limit value was chosen in a way that eliminates unwanted acoustic disturbances in the assembly hall.

The proposed algorithm for the anomaly detection was implemented using the MathWorks MATLAB platform that was also used for the sound analyzes and provides all the required functionalities. These calculations were subsequently integrated into our application, written in the Python programming language. For MATLAB function executions, MATLAB Engine API available for Python was used. Although it is possible to rewrite the functions into the Python programming language, in our proof of concept we decided to utilize MATLAB functionalities directly, for easier integration with the sound analysis phases.

### 4.6. Bearing Failure Detection Visualization

**Figure 15:**
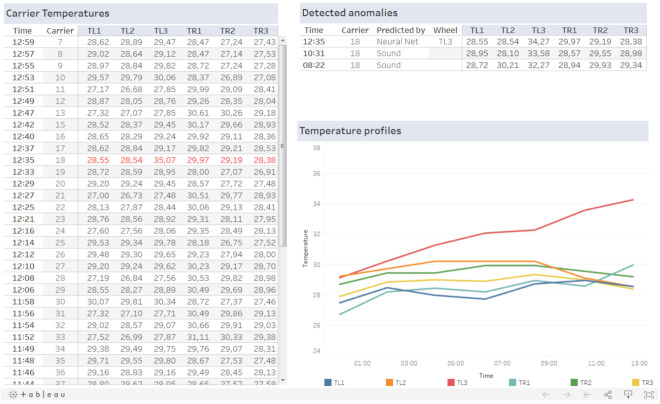
Implemented smart system dashboard for assembly hall maintenance.

To give the maintenance crew a tool for bearing health monitoring, a dashboard was implemented that allows them access to the collected data and anomaly detection results. The visualization was built on top of the ClickHouse database that integrated the data collected from the IIoT devices and stationary temperature measurements. For the identification and prediction of the bearing failure, we utilized the proposed algorithms that labeled identified anomalies in the collected data and created email and SMS notifications to notify the assembly hall maintenance. The example of the implemented dashboard is shown in Figure 15.

The implemented dashboard shows carrier temperature measurements in the last two hours on the left side. Anomalies detected by the implemented trigger-based algorithms based on the thresholds and neural network together with the bearing health monitoring by sound measurements are shown on the upper right side. For the detected carrier, the temperature profile for the last seven carrier rotation is shown on the lower right side.

The dashboard provides an overview of all the important monitored assembly carrier parameters required by the maintenance crew. Together with email and SMS notifications, it provides powerful tool for minimizing assembly conveyor downtime.

## 5. Discussion

The advantage of the IIoT data collection approach is in our case a complex coverage of the measurement of individual devices, during the entire assembly process, which provides opportunities for a more comprehensive analysis of the problem area. It is also suitable for collecting data from constantly moving devices, due to the fact that the operating time is several months or years without external power supply.

A limitation in our case was the mounting of sensors on individual devices. Mechanical intervention on the carriers was not the preferred method of device and sensor mounting. Due to this limitation, the usage of vibrometer devices was not considered for anomaly detection. A complete implementation of IIoT sensors would also require the use of custom-built IIoT devices that would support six temperature sensors, but these are not available as standard.

Despite the suitability of the IIoT data collection approach, it was unacceptable in terms of the plant’s production reliability strategy to install IIoT sensors on each carrier. For this reason, as an alternative solution, we proposed and implemented a stationary temperature and sound measurement infrastructure, which allowed us to measure and record the temperatures and sounds of all carriers in the assembly conveyor.

With this approach, we created a universal, inexpensive, and easy-to-implement solution, where it is possible to measure values from any number of carriers and on any type of assembly line. The disadvantage of this solution is that the availability of the measured temperature information is only every two hours, when the carrier passes through the temperature measurement point on the carrier lift.

From the point of view of bearing failure detection, the implemented solution using trigger-based anomaly detection seems to be a suitable approach. The main advantage was the possibility of a very fast deployment in this approach, given that from the available data it was possible to derive parameters for anomaly detection very soon requiring a small amount of data.

Using this approach, we were able to detect an anomaly, a bearing failure, approximately 2 to 4 h before critical bearing damage during normal operation. A limitation of this solution is the need to manually set the detection parameters to capture only the actual bearing failure while minimizing false detection, which causes confusing notifications to the operator.

Applying a neural network approach allowed us to eliminate the need to manually set parameters for anomaly detection. Based on the available data we were able to predict critical bearing damage using the proposed neural network, based on the currently measured bearing temperatures. This approach was able to predict bearing damage approximately 4 to 6 h before critical damage.

To deploy this approach, it is necessary to have a sufficient amount of data available to learn the neural network, which makes it possible to deploy this approach later.

Both previous approaches detect bearing damage indirectly due to increasing temperature caused by guide line friction.

Therefore, we decided to supplement the anomaly detection with sound analysis, which is possible to predict the bearing state on the carrier in the phase when the limitation of its functionality begins. This can give the maintenance crew time to analyze bearing health before the failure itself and can lead to assembly line modification to mitigate the bearing failure causes. This means that with this approach it is possible to detect bearing state anomaly approximately 8 to 10 h before its critical damage.

The disadvantage of this approach is the impossibility of accurately identifying a particular damage bearing on the carrier due to the fact that the sound is analyzed as a whole.

Although the used methods are calculated and performed over the data store in the SQL storage, there should be no performance limitations implementing them on the used IPC directly performance wise. The anomaly detection, using a trigger-based approach and neural networks, can provide results in near real time. Detection using the sound analysis can provide results in a few seconds that is still a fraction of the time between the individual sample recordings.

Each of these approaches can be used separately, but by appropriately combining these approaches, using their advantages and suppressing their disadvantages, we have proposed and implemented a unique approach that allows more accurate damage prediction and significantly minimizes the possibility of false detection.

While the symbiosis of the neural network and sound analysis are used for prediction well in advance, the identification of damage by trigger-based anomaly detection is a direct starter of the maintenance process.

## 6. Conclusions

Damaged carrier wheel bearing is the most common cause of cessation of the assembly conveyor. Using a proposed analysis of the sound signal, it is possible to predict the failure of a bearing on the carrier in the phase when the limitation of its functionality begins. Subsequently, with the deterioration of bearing damage, its temperature increases. This allows the identification of the specific wheel or guide bearing using our trigger-based detection and neural network approaches.

The implemented smart monitoring system made it possible to predict and detect damage to the bearings of the transport carrier in time at a stage when the operation of the conveyor was not endangered. Alerts are sent to the maintenance department when bearing failure occurs using email and SMS notifications. Thus, a preventive replacement of a damaged bearing could be performed during the next regular scheduled production outage, minimizing production downtime.

The maintenance crew thus received a powerful tool to predict and identify the damaged bearing before its complete seizure. The use of an implemented application platform can prevent an unexpected cessation of the entire assembly process and avoid significant financial losses. Performing the predictive maintenance, based on notifications from the realized application platform, ensures assembly process continuity and compliance with the planned number of produced cars. This is what our proposal has realized.

## Figures and Tables

**Table 1:** Adeunis ARF8180BA—selected technical parameters.

Name	Specification
Type	LoRaWAN transmitter
Radio Frequency (RF)	Frequency: 863–870 MHz
Sensor	Temperature
Antenna	Internal
Maximum range	15,000 m
Operating temperature	−20 to +75 °C
Power	Input voltage: 3.6 V, Battery: Built-in, Li-SOCl2, Battery life: Up to 10 years

**Table 2:** Temperature sensor—selected technical parameters.

Name	Specification
Internal sensor—temperature range	−30 to +70 °C
Internal sensor—Inertia by 10 °C steps	90 min
External sensor—temperature range	−55 to +155 °C
External sensor—Inertia by 10 °C steps	15 min
External sensor—Cable length	2 m
Resolution	0.1 °C
Precision	±0.1 °C

**Table 3:** Data Logger RAY-3—selected technical parameters.

Name	Specification
CPU	CORTEX M4
Ethernet	10/100 Mbps, RJ45, distance up to 100 m
Discrete IN	4 sink contact
Discrete OUT	2 relay 3 A
Analog IN	6 resistance, voltage or current, reading 10 times per second
Analog OUT	2 voltage 0–10 V, load up to 5 mA
Protocols	Modbus RTU, Modbus TCP/IP, IP, ICMP, UDP, TCP, DHCP, PPP, ARP, SNTP, IEC60870-5-104:2000, DynDNS, FTP server, FTP client, DNS client
Power supply	9–36 VDC
Real time clock	Yes

**Table 4:** Calex PC21MT-0—selected technical parameters.

Name	Specification
Temperature Rang	0 °C to 250 °C
Field of View	2:1
Target Temperature Output	4–20 mA
Sensor Temperature Output	Not available
Accuracy	±1% of reading or ±1 °C whichever is greater
Repeatability	±0.5% of reading or ±0.5 °C whichever is greater
Response Time, t_90_	240 ms (90% response)
Spectral Range	8 to 14 μm
Supply Voltage	24 V DC (28 V DC max.)

**Table 5:** Radio-Frequency Identification (RFID) reader SICK RFID RFH630-1000001—selected technical parameters.

Name	Specification
Frequency band	HF (13.56 MHz)
RFID standard	ISO/IEC 15693, ISO/IEC 18000-3 “Mode 1”
Read range	≤240 mm
Typical access times	UID read (64 bits/8 bytes): 18 ms
Connection type	1 x cable, 15-pin D-Sub HD male connector
Supply voltage	10 V DC to 30 V DC
Serial	Yes, RS-232, RS-422, RS-485(RS-422/RS-485 only via 4-wire)

**Table 6:** Industrial PC—selected technical parameters.

Name	Specification
CPU	Intel Core i3-6100U 2C/4T GT2 2.3 GHz 3M/
Cooler	Fanless
System Memory	4 GB, DDR4 2133/1867 MHz, non-ECC, un-buffered memory
Storage Devices	M.2 2280 128 GB SSD
Ethernet	Realtek 8111 G Giga LAN 10/100/1000 Mb LAN × 2, RJ-45 × 2
Audio	Realtek^®^ ALC887
USB	USB3.0 Port × 4
Serial Port	RS-232 Port × 1
WatchDog Timer	1~255 steps by software program

**Table 7:** Microphone miniDSP UMIK-1—selected technical parameters.

Name	Specification
Type	6 mm electret
Polar Patter	Omni-Directional
Frequency response	20–20,000 Hz
Connector	miniUSB, USB Audio class 1.0
Resolution	24 bit ADC
Sample rate	48 kHz
Max SPL for 1% TDH @ 1 kHz	133 dB SPL @OdB gain setting
Output noise level at max IGPA	−74 dBFS

**Table 8:** Correlation matrix of input parameters. TAmb: Ambient temperature.

Parameter	TR1 avg	TL1 avg	TR2 avg	TL2 avg	TR3 avg	TL3 avg	TAmb
TR1 avg	1.0000	0.9456	0.7036	0.6847	0.9817	0.5930	0.8475
TL1 avg	0.9456	1.0000	0.5765	0.6424	0.9654	0.5400	0.8203
TR2 avg	0.7036	0.5765	1.0000	0.8879	0.6859	0.9175	0.8669
TL2 avg	0.6847	0.6424	0.8879	1.0000	0.7038	0.9273	0.8742
TR3 avg	0.9817	0.9654	0.6859	0.7038	1.0000	0.6037	0.8329
TL3 avg	0.5930	0.5400	0.9175	0.9273	0.6037	1.0000	0.8277
TAmb	0.8475	0.8203	0.8669	0.8742	0.8329	0.8277	1.0000

**Table 9:** Part of the obtained neural networks. MLP: MultiLayer Perceptron.

Topology	Train. Perf. [%]	Test. Perf. [%]	Valid. Perf. [%]	Error Func.	Hidden Activ.	Output Activ.
MLP 13-16-2	92.85	91.28	93.71	Sum of squares	Identity	Softmax
MLP 13-18-2	93.28	94.38	94.03	Entropy	Exponential	Softmax
MLP 13-15-2	93.12	94.83	91.44	Entropy	Identity	Logistic
MLP 13-19-2	92.54	91.24	91.86	Sum of squares	Logistic	Identity
MLP 13-11-2	92.11	92.45	91.82	Entropy	Tanh	Exponential

**Table 10:** Validation data set confusion matrix of the selected neural network.

Actual Amount	Estimated Amount		
	Failure	OK	Total
Failure	70	17	87
OK	16	450	466
Total	86	467	553

**Table 11:** Performance evaluation parameters of the selected neural network.

Parameter	Value	Value [%]
Precision	0.8139	81.39
Recall	0.8045	80.45
Accuracy	0.9403	94.03
F_1_ Score	0.8092	80.92

**Table 12:** Bearing “SKF 22206 E” frequencies for different carrier speeds. Rotation per Minute (RPM), Ball Pass Frequency Outer (BPFO), Ball Pass Frequency Inner (BPFI), Ball Spin Frequency (BSF), Fundamental Train Frequency (FTF).

*v* [m/min]	RPM	f_r_ [Hz]	BPFO [Hz]	BPFI [Hz]	BSF [Hz]	FTF [Hz]	f_rp_ [Hz]
8	35.37	0.59	3.9	5.5	1.7	0.25	3.3
9	39.79	0.66	4.4	6.2	1.9	0.28	3.8
10	44.21	0.74	4.9	6.9	2.1	0.31	4.2
11	48.63	0.81	5.4	7.6	2.3	0.34	4.6
12	53.05	0.88	5.9	8.3	2.5	0.37	5.0
13	57.47	0.96	6.4	8.9	2.7	0.40	5.4

## Data Availability

Not applicable.

## Appendix A

This appendix contains examples of Python code used in our implemented application platform. Custom ALSA error handler required for sound recording is shown in Figure A1.

Example of trigger-based anomaly detection configuration used in our implemented application platform is shown in Figure A2.

## References

[1] Ahuett-Garza H., Kurfess T. (2018). A brief discussion on the trends of habilitating technologies for Industry 4.0 and Smart manufacturing. Manuf. Lett..

[2] Frank A.G., Dalenogare L.S., Ayala N.F. (2019). Industry 4.0 technologies: Implementation patterns in manufacturing companies. Int. J. Prod. Econ..

[3] Lu Y. (2017). Industry 4.0: A survey on technologies, applications and open research issues. J. Ind. Inf. Integr..

[4] Algabroun H., Iftikhar U., Al-Najjar B., Weyns D. (2018). Maintenance 4.0 framework using self: Adaptive software architecture. J. Maint. Eng..

[5] Lima E., Gorski E., Loures E.F., Santos E.A.P., Deschamps F. Applying machine learning to AHP multicriteria decision making method to assets prioritization in the context of industrial maintenance 4.0. Proceedings of the 9th IFAC Conference on Manufacturing Modelling, Management and Control MIM 2019.

[6] What is Maintenance 4.0?. https://valuekeep.com/resources/e-books-articles/what-is-maintenance-4-0/.

[7] What is Maintenance 4.0?. https://gesrepair.com/what-is-maintenance-4-0/.

[8] Jasiulewicz—Kaczmarek M., Gola A. (2019). Maintenance 4.0 Technologies for Sustainable Manufacturing—An Overview. IFAC-PapersOnLine.

[9] Bousdekis A., Lepenioti K., Apostolou D., Mentzas G. Decision making in Predictive Maintenance: Literature Review and Research Agenda for Industry 4.0. Proceedings of the 9th IFAC Conference on Manufacturing Modelling, Management and Control MIM 2019.

[10] Kimera D., Nduvu Nangolo F. (2020). Predictive maintenance for ballast pumps on ship repair yards via machine learning. Transp. Eng..

[11] Kamat P., Sugandhi R. (2020). Anomaly Detection for Predictive Maintenance in Industry 4.0—A survey. EVF’2019, E3S Web of Conferences 170, 02007. www.e3s-conferences.org/articles/e3sconf/pdf/2020/30/e3sconf_evf2020_02007.pdf.

[12] Erhan L., Ndubuaku M., Di Mauro M., Song W., Chen M., Fortino G., Bagdasar O., Liotta A. (2021). Smart anomaly detection in sensor systems: A multi-perspective review. Inf. Fusion.

[13] Carletti M., Masiero C., Beghi Gian A., Susto A. (2019). A deep learning approach for anomaly detection with industrial time series data: A refrigerators manufacturing case study. Procedia Manuf..

[14] Lindemanna B., Fesenmayr F., Jazdi N., Weyrich M. (2019). Anomaly detection in discrete manufacturing using self-learning approaches. Procedia CIRP.

[15] Folmer J., Vogel-Heuser B. (2012). Model-based Approach to Generate Training Sequences for Discrete Event Anomaly Detection in Manufacturing. Ifac Proc. Vol..

[16] What is Maintenance 4.0?. https://reliabilityweb.com/what-is-maintenance-4.0.

[17] Srivastava P., Shukla R.K., Sharma S., Khanduja D., Gupta R., Alrasheedi M., Singh G. Fuzzy Methodology Approach for Prioritizing Maintenance 4.0 Attributes. Proceedings of the 2020 International Conference on Computation, Automation and Knowledge Management (ICCAKM).

[18] Cachada A., Moreira P.M., Romero L., Barbosa J., Leitno P., Gcraldcs C.A.S., Deusdado L., Costa J., Teixeira C., Teixeira J. Maintenance 4.0: Intelligent and Predictive Maintenance System Architecture. Proceedings of the 2018 IEEE 23rd International Conference on Emerging Technologies and Factory Automation (ETFA).

[19] Câmara R.A., Mamede H.S., dos Santos V.D. Predictive Industrial Maintenance with a Viable Systems Model and Maintenance 4.0. Proceedings of the 2019 8th International Conference on Software Process Improvement (CIMPS).

[20] Cachada A., Barbosa J., Leitão P., Alves A., Alves L., Teixeira J., Teixeira C. Using Internet of Things Technologies for an Efficient Data Collection in Maintenance 4.0. Proceedings of the 2019 IEEE International Conference on Industrial Cyber Physical Systems (ICPS).

[21] Liu Y., Pang Z., Karlsson M., Gong S. (2020). Anomaly detection based on machine learning in IoT-based vertical plant wall for indoor climate control. Build. Environ..

[22] Evangelou M., Adams N.M. (2020). An anomaly detection framework for cyber-security data. Comput. Secur..

[23] Dybkowski M., Klimkowski K. (2019). Artificial Neural Network Application for Current Sensors Fault Detection in the Vector Controlled Induction Motor Drive. Sensors.

[24] Tang T.-W., Kuo W.-H., Lan J.-H., Ding C.-F., Hsu H., Young H.-T. (2020). Anomaly Detection Neural Network with Dual Auto-Encoders GAN and Its Industrial Inspection Applications. Sensors.

[25] Francik S., Kurpaska S. (2020). The Use of Artificial Neural Networks for Forecasting of Air Temperature inside a Heated Foil Tunnel. Sensors.

[26] Liu J., Wang P., Jiang D., Nan J., Zhu W. (2020). An integrated data-driven framework for surface water quality anomaly detection and early warning. J. Clean. Prod..

[27] Oucheikh R., Fri M., Fedouaki F., Hain M. (2020). Deep Real-Time Anomaly Detection for Connected Autonomous Vehicles. Procedia Comput. Sci..

[28] Lughofer E., Zavoianu A.-C., Pollak R., Pratama M., Meyer-Heye P., Zörrer H., Eitzinger C., Radauer T. (2020). Online anomaly detection with advanced independent component analysis of multi-variate residual signals from causal relation networks. Inf. Sci..

[29] Poor P., Basl J., Zenisek D. Predictive Maintenance 4.0 as next evolution step in industrial maintenance development. Proceedings of the 2019 International Research Conference on Smart Computing and Systems Engineering (SCSE).

[30] Einabadi B., Baboli A., Ebrahimi M. Dynamic Predictive Maintenance in industry 4.0 based on real time information: Case study in automotive industries. Proceedings of the 9th IFAC Conference on Manufacturing Modelling, Management and Control MIM 2019.

[31] Carvalho T.P., Soares F.A., Vita R., Francisco R.D.P., Basto J.P., Alcalá S.G. (2019). A systematic literature review of machine learning methods applied to predictive maintenance. Comput. Ind. Eng..

[32] Nguyen T.P., Medjaher K. (2019). A new dynamic predictive maintenance framework using deep learning for failure prognostics Khanh. Reliab. Eng. Syst. Saf..

[33] Sahal R., Breslin J.G., Ali M.I. (2020). Big data and stream processing platforms for Industry 4.0 requirements mapping for a predictive maintenance use case. J. Manuf. Syst..

[34] Zonta T., Da Costa C.A., Righi R.D.R., De Lima M.J., Da Trindade E.S., Li G.P. (2020). Predictive maintenance in the Industry 4.0: A systematic literature review. Comput. Ind. Eng..

[35] Dalzochio J., Kunst R., Pignaton E., Binotto A., Sanyal S., Favilla J., Barbosa J. (2020). Machine learning and reasoning for predictive maintenance in Industry 4.0: Current status and challenges. Comput. Ind..

[36] Uckelmann D., Harrison M., Michahelles F., Uckelmann D., Harrison M., Michahelles F. (2011). An Architectural Approach to-wards the Future Internet of Things. Architecting the Internet of Things.

[37] Kerlink–Wirnet Station. https://www.kerlink.com/product/wirnet-station/.

[38] Adeunis—Test IoT network Coverage. https://www.adeunis.com/en/produit/ftd-network-tester/.

[39] Adeunis—Temp: IoT temperature reading. https://www.adeunis.com/en/produit/temp-temperature/.

[40] Viltrus Electronics—Data Logger RAY-3. http://www.viltrus.com/data-logger-ray-3/.

[41] Optris—Pyrometer Optris CS LT. https://www.optris.global/optris-cs-lt-csmed-lt.

[42] Calex Electronics Limited—PyroCouple—Simple Infrared Temperature Sensor with Analogue Output. https://www.calex.co.uk/product/temperature-measurement/infrared-temperature-sensors/pyrocouple/.

[43] Sick-RFID-RFH6xx/RFH630. https://cdn.sick.com/de/en/identification-solutions/rfid/rfh6xx/rfh630-1000001/p/p258850.

[44] Aaeon–NANO-002N. https://www.aaeon.com/en/p/turn-key-chassis-solutions-nano-002n.

[45] MiniDSP–UMIK-1. https://www.minidsp.com/products/acoustic-measurement/umik-1.

[46] Tandon N., Nakra B.C. (1992). Vibration and acoustic monitoring techniques for the detection of defects in rolling element bearings—A review. Shock Vib. Dig..

[47] Tandon N., Choudhury A. (1999). A review of vibration and acoustic measurement methods for the detection of defects in rolling element bearings. Tribol. Int..

[48] Carden E.P., Fanning P. (2004). Vibration based condition monitoring: A review. Struct. Health Monit..

[49] Rai A., Upadhyay S.H. (2016). A review on signal processing techniques utilized in the fault diagnosis of rolling element bearings. Tribol. Int..

[50] Zhen D., Guo J., Xu Y., Zhang H., Gu F. (2019). A Novel Fault Detection Method for Rolling Bearings Based on Non-Stationary Vibration Signature Analysis. Sensors.

[51] Karacay T., Akturk N. (2009). Experimental diagnostics of ball bearings using statistical and spectral methods. Tribol. Int..

[52] Archambault J., Archambault R., Thomas M. (2002). Time domain descriptors for rolling-element bearing fault detection. Proceedings of the 20th Seminar on Machinery Vibration, Canadian Machinery Vibration Association.

[53] Dron J.P., Bolaers F., Rasolofondraibe L. (2004). Improvement of the sensitivity of the scalar indicators (crest factor, kurtosis) using a de-noising method by spectral subtraction: Application to the detection of defects in ball bearings. J. Sound Vib..

[54] Antoni J. (2006). The spectral kurtosis: A useful tool for characterizing non-stationary signals. Mech. Syst. Signal Process..

[55] Kim S., An D., Choi J.-H. (2020). Diagnostics 101: A Tutorial for Fault Diagnostics of Rolling Element Bearing Using Envelope Analysis in MATLAB. Appl. Sci..

[56] Shi S., Randall R., Antoni J. Rolling element bearing fault detection using improved envelope analysis. Proceedings of the 8th International Conference on Vibrations in Rotating Machinery.

[57] Dyer D., Stewart R.M. (1978). Detection of rolling element bearing damage by statistical vibration analysis. Trans. ASME J. Mech. Des..

[58] Taylor J.I. (1980). Identification of bearing defects by spectral analysis. J. Mech. Des..

[59] Orhan S., Akturk N., Celik V. (2006). Vibration monitoring for defect diagnosis of rolling element bearings as a predictive maintenance tool: Comprehensive case studies. Ndt E Int..

[60] Randalla R.B., Antoni J. (2011). Rolling element bearing diagnostics—A tutorial. Mech. Syst. Signal Process..

[61] McFadden P.D., Smith J.D. (1984). Vibration monitoring of rolling element bearings by the high-frequency resonance technique—A review. Tribol. Int..

[62] Shea J.M., Taylor J.K. (1992). Spike energy in faults analysis machine condition monitoring. Noise Vib. World-Wide.

[63] De Priego J.C.M. (2001). The relationship between vibration spectra and spike energy spectra for an electric motor bearing defect. Vibrations.

[64] Yoshioka T., Fujiwara T. (1984). Application of acoustic emission technique to detection of rolling bearing failure. Am. Soc. Mech. Eng..

[65] Holroyd T. Condition monitoring of very slowly rotating machinery using AE techniques. Proceedings of the 14th International Congress on Condition Monitoring and Diagnostic Engineering Management.

[66] Al-Ghamd A.M., Mba D. (2006). A comparative experimental study on the use of acoustic emission and vibration analysis for bearing defect identification and estimation of defect size. Mech. Syst. Signal Process..

[67] Entezami M., Stewart E., Tutcher J., Driscoll W., Ellis R., Yeo G., Zhang Z., Roberts C., Kono T., Bayram S. Acoustic analysis techniques for condition monitoring of roller bearings. Proceedings of the 6th IET Conference on Railway Condition Monitoring.

[68] Graney B.P., Starry K. (2011). Rolling Element Bearing Analysis. Mater. Eval..

[69] Kebisek M., Tanuska P., Spendla L., Kotianova J., Strelec P. (2020). Artificial Intelligence Platform Proposal for Paint Structure Quality Prediction within the Industry 4.0 Concept. IFAC Papers OnLine.

[70] Spendla L., Kebisek M., Tanuska P., Hrcka L. Concept of predictive maintenance of production systems in accordance with industry 4.0. Proceedings of the SAMI 2017—IEEE 15th International Symposium on Applied Machine Intelligence and Informatics.

[71] Vazan P., Janikova D., Tanuska P., Kebisek M., Cervenanska Z. (2017). Using data mining methods for manufacturing process control. IFAC-PapersOnLine.

[72] Foody G.M. (2017). Impacts of Sample Design for Validation Data on the Accuracy of Feedforward Neural Network Classification. Appl. Sci..

[73] May R.J., Maier H.R., Dandy G.C. (2010). Data splitting for artificial neural networks using SOM-based stratified sampling. Neural Netw..

[74] Arnab R. (2017). Survey Sampling Theory and Applications.

[75] Harris C.M., Piersol A.G. (2002). Shock and Vibration Handbook.

[76] Sassi S., Badri B., Thomas M. “TALAF” and “THIKAT” as innovative time domain indicators for tracking ball bearings. Proceedings of the 24th Seminar on Machinery Vibration.

